# The association between serum 25-hydroxyvitamin D3 concentration and serum lipids in the rural population of China

**DOI:** 10.1186/s12944-017-0603-6

**Published:** 2017-11-14

**Authors:** Huina Ge, Hualei Sun, Teng Wang, Xinxin Liu, Xing Li, Fei Yu, Han Han, Jun Wang, Wenjie Li

**Affiliations:** Department of Nutrition and Food Hygiene, College of Public Health, Zhengzhou University, Henan, 450001 China

**Keywords:** 25(oh)D3, Dyslipidemia, Serum lipid

## Abstract

**Background:**

Vitamin D deficiency is implicated in some diseases, including cardiovascular. Few studies have assessed the correlation between 25-hydroxyvitamin D3 [25(OH) D3] and serum lipids. In this study, we explored the correlation between serum 25(OH) D3 concentrations and serum lipids with a typical sample of the rural population in China.

**Methods:**

Face-to-face research was used to gather some basic information. Fasting serum concentrations of 25-(OH) D3, total cholesterol (TC), triglyceride (TG), HDL cholesterol (HDL-C) and, LDL cholesterol (LDL-C) tested in the laboratory.

**Results:**

The mean of serum 25(OH) D3 level was 28.71 ± 29.29 ng/mL. The results showed that the dyslipidemia was strongly associated with gender (*P* = 0.031), drinking (*P* = 0.043), high-fat diet intake (*P* = 0.017), HDL-C (*P*<0), TG (*P*<0), body mass index (BMI) (*P*<0) and serum 25(OH)D3 levels (*P* = 0.002). There was a positive correlation between serum 25(OH)D3 and HDL-C (*P*<0) in all groups. The relationship between 25(OH) D3 and LDL-C (*P* = 0.024) was discovered only in normal lipid group. The multivariable adjusted odds ratio (95%CI) of hypoalphalipoproteinemia/HDL and dyslipidemia by comparing the sufficiency vs. the deficiency serum 25-(OH) D3 level were 0.31 (0.192, 0.499) (*P* = 0.001) and 0.52 (0.36, 0.73) (*P* = 0.005), respectively.

**Conclusions:**

Serum 25(OH) D3 concentrations were associated with the serum lipids level and the association was different in normal serum lipid group and dyslipidemia group. With the increase of serum 25(OH) D3 levels, the incidence of dyslipidemia decreased.

## Background

Vitamin D is advised to have many functions such as regulating calcium homeostasis and bone metabolism, adjusting immune function, decreasing insulin resistance and anti-inflammatory effect [1–4]. Vitamin D could be obtained from diet including some types of fish, cereals, dairy products and vitamin D supplements [5]. The main way of obtaining vitamin D is getting enough sun exposure. When the skin is exposed to solar UV radiation, 7-dehydrocholesterol can be transformed to previtamin D3. Previtamin D3 could be hydroxylated into 25-hydroxyvitamin D3 [25(OH) D] in the liver and then hydroxylated into 1, 25-dihydroxyvitamin D [1, 25(OH)_2_ D] in the kidney [6, 7]. Vitamin D deficiency has become a pandemic, furthermore it is an under-diagnosed and under-treated vitamin deficiency [8, 9]. Vitamin D deficiency is also indiscriminate in individuals without respect to their age, race and geography. The serum concentration of 25-hydroxyvitamin D >30 ng/mL is sufficiency and can realize the vitamin D’ s beneficial effects maximization for health. When the sun exposure is insufficiency, the children and adults need to intake at least 800–1000 IU vitamin D_3_/day [10].

Dyslipidemia including hypercholesterolemia and hypertriglyceridemia causes a serious danger to people’s health in universal population. It is also an important risk factor for the occurrence of cerebral ischemic and coronary heart disease [11, 12]. A recent study has shown that vitamin D insufficiency evidently causes the mechanism of esophageal, renal, lung, gastric, endometrial cancer and non-Hodgkin’s lymphoma [13]. Vitamin D deficiency is associated with elevated levels of some biomarker of cardiovascular risk in a United States population [14]. There was a correlation between vitamin D deficiency and the increasing serum levels of TC and LDL-C, as well as the decreasing serum levels of HDL-C [15–20]. However, the conclusions are inconsistent and relationships often disappeared after adjusting for confounding factors [17, 18, 20, 21].

The purpose of this study was to analyze the relationships of 25-hydroxyvitamin D3 [25(OH) D3] concentrations and serum lipid including serum level of TC, TG, HDL-C, LDL-C and with the incidence of dyslipidemia in rural population of China.

## Methods

### Study design and population

**Fig. 1:**
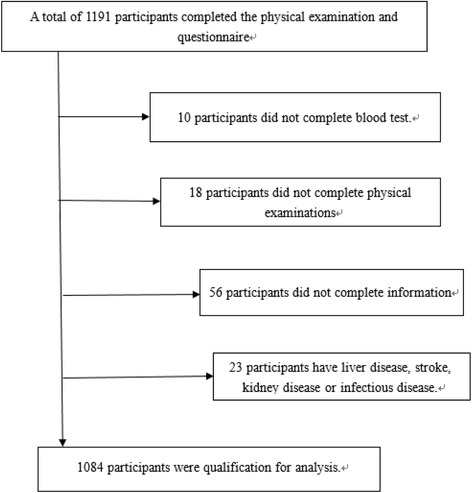
Flow chart of participant incorporation

Participants were collected from a cross-sectional study in three different arears, including Wuzhi, Xin’an and Houzhai county of Henan Province in China from July 2013 to August 2015. The acceptance criteria to participate included recruitment between aged of 18–79 years, lack of liver disease, stroke, kidney disease, and infectious disease. A total of 1191 participants completed the physical examination and questionnaire, but only 1084 participants conform to the acceptance criteria (Fig.1).

### Data collection and laboratory measurements

Anthropometric measurements were performed according to normative procedures. Height and weight (without outdoor wear and shoes) were measured to the nearest 0.1 cm and 0.1 kg using an electronic scale. Body mass index (BMI) was acquired by the formula: BMI = weight (kg)/height (m^2^). All demographic data were obtained by face to face investigation. The questionnaire was accomplished by well-trained interviewers, containing information on demographic characteristics (gender, age, name, education level, occupation, *et,al*),life styles (smoking, alcohol drinking, drinking tea, intakes of vegetable, fruit and fat, *et,al*), the individual history of disease (stroke, liver disease, kidney disease, infectious disease, *et,al*).

### Blood samples

Fasting venous blood samples collection (10 mL) were accomplished between 6:00 am and 9:00 am by well-trained nurse after at least 8 h of fasting. Blood samples were collected with a vacuum blood tube and centrifuged with 3000 rpm for 15 min, aliquoted and stored at −80 °C until further using.

### Laboratory measurements

Serum 25(OH) D3 was a better indicator of vitamin D status than 1,25(OH)_2_D3 [22]. Serum concentration of 25(OH) D3 was measured by an enzyme-linked immunosorbent assay (ELISA) according to the product specifications [Sangon Biotech (Shanghai) Co., Ltd., China]. The microplate reader (BIO-RAD680, USA) was used to measure the absorbance (450 nm). The stronger of the color, the lower concentrations of the 25(OH) D3. The inter-assay CV of the total process was ≤9.9% for 25(OH) D3. The serum status of TC, TG, HDL-Ctested by biochemistry analyzer (KHB360, Shanghai, China). The detection method of TC, TG, HDL-C and LDL-C were GPO-PAP, GHOD-PAP, direct method of catalase clearance and direct method of surfactant removal, respectively.

### Diagnostic criteria

In this study the serum 25(OH) D3 levels<20 ng/mL were confirmed as vitamin D deficiency. 25(OH) D3 levels were insufficient between 20 ng/mL to 30 ng/mL and 25(OH) D3 status>30 ng/mL was sufficiency [23, 24]. The category of TC, TG, HDL-C and LDL-C were showed in Table 1 [25]. The diagnostic criteria of dyslipidemia are defined according to serum lipids status. The cut-off values for higher triglyceride, higher cholesterol, lower high density lipoprotein and higher low density lipoprotein were 2.26 mmol/L, 6.22 mmol/L, 1.04 mmol/L and 4.14 mmol/L [12].

**Table 1:** The category of Total Cholesterol, Triglycerides Level, HDL Cholesterol and LDL Cholesterol level

Variable	Desirable	Borderline high/low	High/Low
Total Cholesterol Level	Less than 5.18 mmol/L	5.18–6.19 mmol/L	6.22 mmol/dl and above
Triglycerides Level	Less than 1.70 mmol/L	1.70–2.26 mmol/L	2.26 mmol/dl and above
LDL Cholesterol Level	Less than 3.37 mmol/L	3.37–4.13 mmol/L	4.14 mmol/dl and above
HDL Cholesterol Level	greater than 1.55 mmol/L	1.04–1.55 mmol/L	Less than 1.04 mmol/dl

### Statistical analysis

Participants were divided into two groups according to serum lipid levels, including normal lipid group and dyslipidemia group. The participant in the case group and the control group were divided into three groups according to the serum 25(OH) D3 level, including vitamin D deficiency group [25(OH) D3<20 ng/mL], vitamin D insufficiency group [20 ng/mL ≤ 25(OH) D3<30 ng/mL] and vitamin D sufficiency group [25(OH) D3 ≥ 30 ng/mL]. The characteristics of participants were presented as relative frequency (%) for categorical variables. The characterization of continuous variables was presented as means and standard deviation (SD). The chi square test was used to compare the differences of categorical variables and continuous variables using One-way analysis of variance (ANOVA) and t test (normally continuous variables).

Adjusted odds ratios for dyslipidemia status and serum lipid levels (TG, TC, HDL-C and LDL-C) comparing each groups of serum 25(OH) D3 to the lowest serum 25(OH) D3 levels group were analyzed using logistic regression. We used 2 models to adjust the confounding factors. Sex, age and education (high school and above, below high school) were adjusted in model 1. Smoking (never, former, current), drinking (yes, no), high-fat diet (a litter or no, greater than or equal to 25 g/day), tea (yes, no) and vegetables and fruits (a little or no, greater than or equal to 500 g/day) were adjusted in model 2.

We also estimated the interactions between serum 25(OH) D3 levels and gender, education, smoking status, alcohol drinking, vegetables and fruits, tea and high-fat diet. The statistical analyses were carried out by SPSS 17.0 software package (SPSS Inc., Chicago, IL, USA) and STATA version 11.0 (STATA Corp, College Station, Texas, USA). All *p* values were two-sided and <0.05 were considered to be statistically significant.

### Ethics statement

The protocol this research was consented by the Ethics Committee of the Zhengzhou University. All participants were asked to sign the protocol.

## Results

### General characteristics of study participants

The mean of serum 25(OH) D3 levels in the study population was 28.71 ng/mL. The overall prevalence of dyslipidemia was 41.79%. Participants with dyslipidemia were more likely to be male (*P* = 0.031), and to have a higher body mass index (*P*<0), lower serum 25(OH) D3 concentrations (*P*<0), higher fat intake (*P* = 0.017), higher triglycerides levels (*P*<0) and lower HDL cholesterol levels (*P*<0) (Table 2).

**Table 2:** Characteristics of the study population by dyslipidemia status

Variable	No event	Event(dyslipidemia)	
	(*N* = 631)	(*N* = 453)	*P*-value
Age (years)	59.88 ± 12.38	59.53 ± 11.28	0.635
Gender (%)			0.031
Female	62.8	57.0	
Male	37.2	43.0	
Education (%)			0.063
Less than high school	86.6	89.8	
High school or higher	13.4	10.2	
Smoking status (%)			0.358
Current	18.7	18.5	
Former	7.3	9.7	
Never	74.0	71.7	
Drinking (%)			0.043
No	82.0	86.8	
Yes	18.0	13.2	
Vegetables and Fruits (%)			0.051
A little or no	75.6	70.2	
More(≥500 g/days)	24.4	29.8	
Tea (%)			0.132
No	89.0	85.9	
Yes	11.0	14.1	
High-fat diet (%)			0.017
A little or no	84.3	78.6	
More (≥25 g/days)	15.7	21.4	
LDL-C(mmol/L)	2.66 ± 0.66	2.60 ± 0.94	0.236
HDL-C(mmol/L)	1.39 ± 0.26	1.06 ± 0.29	<0
TG(mmol/L)	1.19 ± 0.49	2.15 ± 0.95	<0
TC(mmol/L)	4.59 ± 0.77	4.63 ± 1.17	0.488
BMI(kg/m2)	24.90 ± 3.60	26.18 ± 3.68	<0
25(OH)D3 (ng/mL)	31.03 ± 32.60	25.48 ± 23.57	0.002

*LDL-C* low-density lipoprotein, *HDL-C* high-density lipoprotein, *TG* triglycerides, *TC* total cholesterol, *BMI* body mass index, *25(OH) D3* 25-hydroxyvitamin D3.Values are study weighted means and standard deviation or percentages for continuous or categorical variables, respectively

### The correlation between serum 25(OH) D3 concentrations and serum lipids

**Fig. 2:**
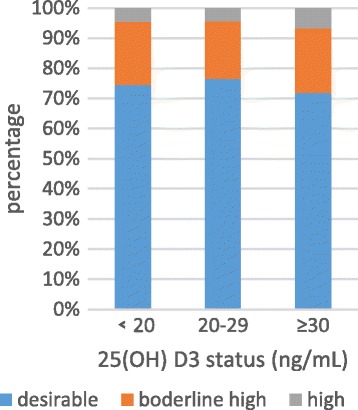
The proportion of serum TC levels according to the 25(OH) D3 level

**Fig. 3:**
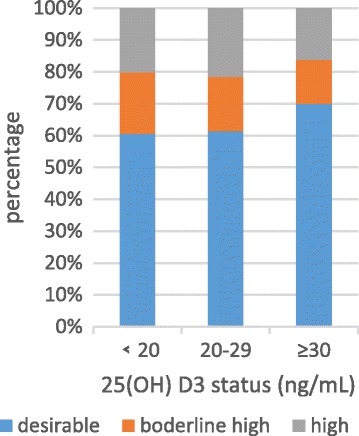
The proportion of serum TG levels according to the 25(OH) D3 level

**Fig. 4:**
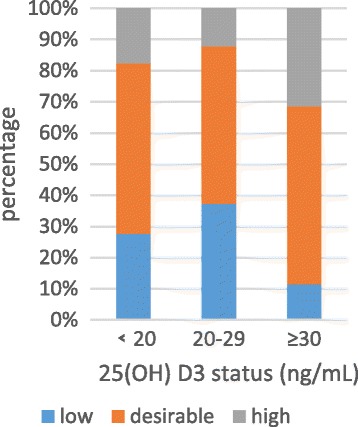
The proportion of serum HDL-C levels according to the 25(OH) D3 level, *P*_trend_ < 0.000

**Fig. 5.**
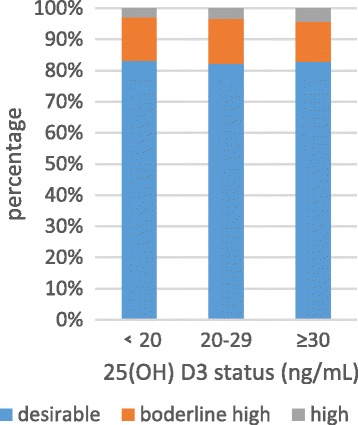
The proportion of serum LDL-C levels according to the 25(OH)D3 level

Anthropometric characteristics and clinical outcome of all participants were presented in Table 2. Among the 1084 participants (430 men and 654 women) and the mean age was 59.7 years old. The overall percentage of vitamin D deficiency, vitamin D insufficiency and vitamin D sufficiency were 53.1%, 29.5% and 19.4%, respectively. Serum 25(OH) D3 levels were positively related to HDL-C (*P*<0) in normal serum lipid group and dyslipidemia group. The negative relationship founded between the serum 25(OH) D3 level and LDL-C (*P* = 0.024) in normal serum lipid group (Table 3). The unadjusted odds ratio (95% CI) and multivariable adjusted odds ratio (95% CI) for higher triglyceride, higher cholesterol, higher low density lipoprotein and lower high density lipoprotein comparing the vitamin D sufficient to the vitamin D deficiency were presented in Table 3. After adjusting for sex, age and education, the odds ratio (95%) for hypobetalipoproteinemia/HDL decreased obviously in vitamin D sufficient compare to vitamin D deficient. The multivariable adjusted OR (95%) for hypoalphalipoproteinemia/HDL comparing the vitamin D sufficient to the vitamin D deficiency was 0.31(0.192, 0.499) (*P* = 0.001) (Table 4). Figures 2, 3, 4 and 5 presented **t**he proportion of serum TC, TG, HDL-C and LDL-C levels according to the 25(OH) D3 level. We could observe from the Figs. 3 and 4 that the proportion of desiable triglycerides level and HDL cholesterol level increased with the increasing of serum 25(OH) D3 levels. Obviously, the information suggested that serume 25(OH) D3 concentrations were associated with blood lipid.

**Table 3:** The correlation between serum 25(OH) D3 concentrations and serum lipids

Variable	Serum 25(OH)D3 Levels							
	<20 ng/mL	20 ng/mL-30 ng/mL	≥30 ng/mL	*P*-value	<20 ng/mL	20 ng/mL-30 ng/mL	≥30 ng/mL	*P*-value
	No event				Event(dyslipidemia)			
	(*N* = 331)	(*N* = 150)	(N = 150)		(*N* = 245)	(*N* = 148)	(*N* = 60)	
LDL-C (mmol/L)	2.68 ± 0.67	2.78 ± 0.69	2.56 ± 0.67	0.024	2.58 ± 0.91	2.62 ± 0.84	2.62 ± 1.22	0.920
HDL-C (mmol/L)	1.39 ± 0.25	1.34 ± 0.24	1.47 ± 0.28	<0	1.06 ± 0.30	0.98 ± 0.21	1.24 ± 0.34	<0
TG (mmol/L)	1.21 ± 0.50	1.23 ± 0.46	1.11 ± 0.48	0.084	2.14 ± 0.95	2.11 ± 0.91	2.29 ± 1.01	0.457
TC (mmol/L)	4.60 ± 0.76	4.65 ± 0.76	4.52 ± 0.82	0.358	4.61 ± 1.16	4.56 ± 1.01	4.89 ± 1.55	0.171
BMI (kg/m^2^)	24.96 ± 3.73	24.80 ± 3.42	24.81 ± 3.48	0.891	26.14 ± 3.84	25.84 ± 3.45	27.19 ± 3.47	0.055
25(OH)D3 (ng/mL)	14.98 ± 2.87	23.96 ± 2.79	73.31 ± 44.97		15.39 ± 3.06	23.65 ± 2.59	71.22 ± 40.53

**Table 4:** Adjusted odds ratios (95%CI) for the presence of hypercholesterolemia, hypertriglyceridemia, hypoalphalipoproteinemia/HDL and hypobetalipoproteinemia/LDL comparing the two highest 25(OH) D3 levels to the deficiency of serum 25(OH) D3 levels, respectively

	Serum 25(OH)D3 Levels			*P*-value
	<20 ng/mL	20 ng/mL-30 ng/mL	≥30 ng/mL	
Hypercholesterolemia (%)	53.3	25.4	21.4	
Unadjusted	1	0.896	1.14	0.644
	(Reference)	(0.646,1.242)	(0.800,1.625)	
Model 1^a^	1	0.922	1.204	0.447
	(Reference)	(0.663,1.282)	(0.840,1.724)	
Model 2^b^	1	0.921	1.193	0.477
	(Reference)	(0.659,1.286)	(0.830,1.714)	
Hypertriglyceridemia (%)	56	28.4	15.6	
Unadjusted	1	0.966	0.659	0.029
	(Reference)	(0.725,1.287)	(0.469,0.925)	
Model 1^a^	1	0.809	0.842	0.246
	(Reference)	(0.584, 1.120)	(0.573,1.237)	
Model 2^b^	1	0.793	0.843	0.239
	(Reference)	(0.570,1.104)	(0.572,1.241)	
Hypoalphalipoproteinemia/HDL (%)	54.2	37.6	8.1	
Unadjusted	1	1.543	0.335	0.002
	(Reference)	(1.146,2.078)	(0.211,0.533)	
Model 1^a^	1	1.527	0.317	0.001
	(Reference)	(1.131,2.061)	(0.199,0.506)	
Model 2^b^	1	1.483	0.31	0.001
	(Reference)	(1.092,2.013)	(0.192,0.499)	
Hypobetalipoproteinemia/LDL (%)	47.2	27.8	25	
Unadjusted	1	1.142	1.474	0.368
	(Reference)	(0.516,20,525)	(0.647,3.361)	
Model 1^a^	1	1.186	1.55	0.308
	(Reference)	(0.534,2.632)	(0.675,3.556)	
Model 2^b^	1	1.086	1.54	0.348
	(Reference)	(0.482,2.446)	(0.663,3.576)	

^a^Model 1. Adjusted for sex, age and education (high school or higher vs. less than high school)

^b^Model 2. Further adjusted for smoking (current, former, never), drinking (yes, no), high-fat diet (a little or no, greater than or equal to 25 g/day), vegetables and fruits (a little or no, greater than or equal to 500 g/day), tea (yes, no)

### The relationships between the occurrences of dyslipidemias and serum 25(OH) D3 concentrations

**Fig. 6:**
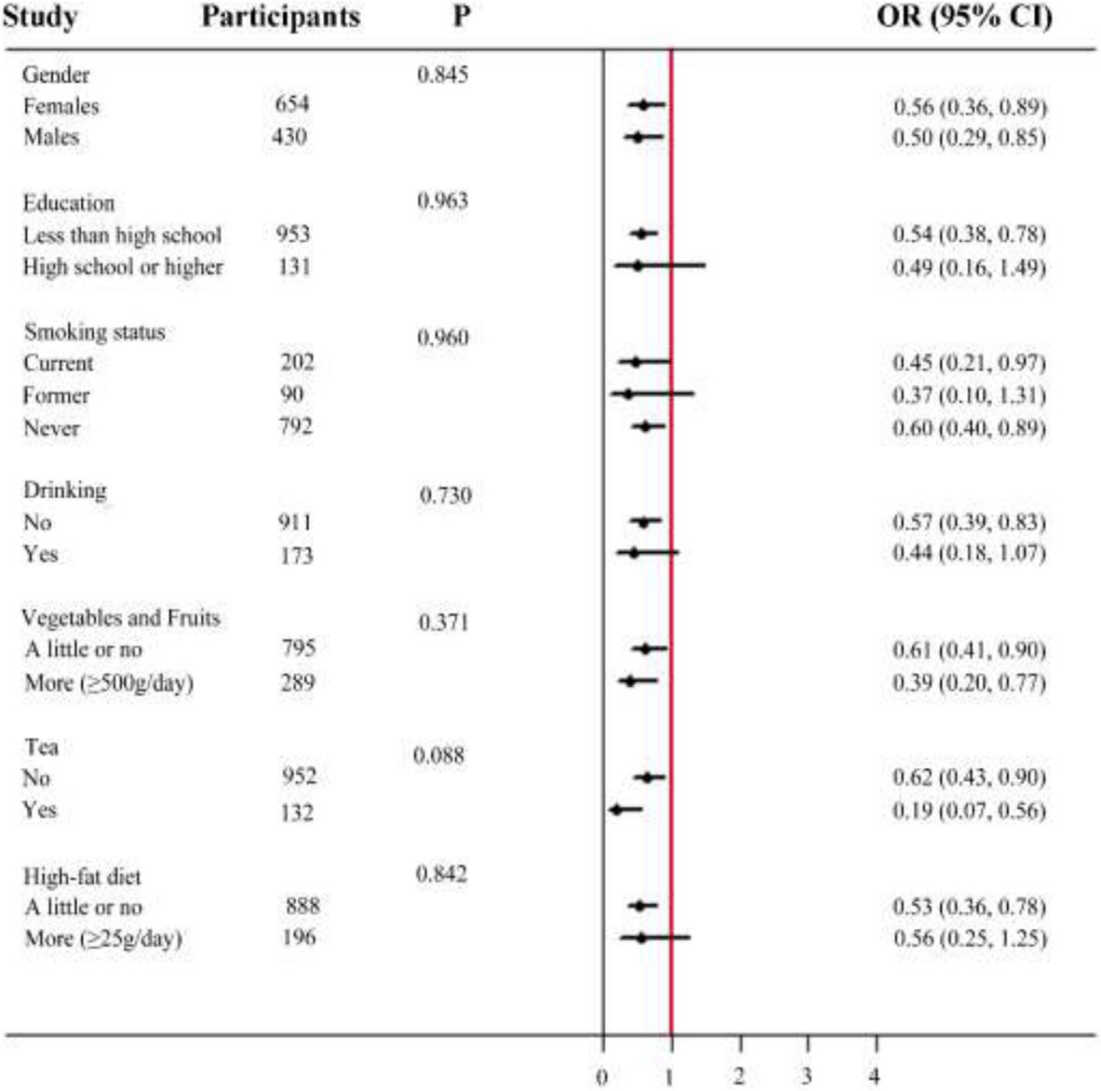
Adjusted odds ratios (95% CI) for dyslipidemia comparing the serum vitamin D sufficient vs serum vitamin D deficient

The unadjusted odds ratio (95% CI) and multivariable adjusted for dyslipidemia comparing the vitamin D sufficient to the vitamin D deficiency were presented in Table 5. After adjusting for sex, age and education, the odds ratio (95%) for dyslipidemias decreased obviously in vitamin D sufficient compare to vitamin D deficient. The multivariable adjusted OR (95%) for dyslipidemia comparing the vitamin D sufficient to the vitamin D deficiency was 0.52 (0.36, 0.73). Figure 6 showed adjusted odds ratios (95% CI) for dyslipidemia comparing the serum vitamin D sufficient vs serum vitamin D deficient, summarizing the association between serum lipid and 25(OH) D3 concentrations. In addition, with the decreasing concentration of 25(OH) D3, the increase in the prevalence of dyslipidemia was linear (*F*-value = 4.65; *p* = 0.031 for trend), indicating a significant relationship.

**Table 5:** Adjusted odds ratios (95%CI) for the presence of dyslipidemia comparing the two highest 25(OH) D3 levels to the deficiency of serum 25(OH) D3 levels

	Serum 25(OH)D3 Levels			
	<20 ng/mL	20 ng/mL-30 ng/mL	≥30 ng/mL	*P*-value
Dyslipidemia (%)	54.08	32.67	13.24	
Unadjusted	1.00	1.33	0.54	0.012
	(Reference)	(1.01,1.77)	(0.38,0.76)	
Model 1^a^	1.00	1.32	0.52	0.006
	(Reference)	(0.99,1.75)	(0.37,0.73)	
Model 2^b^	1.00	1.26	0.52	0.005
	(Reference)	(0.95,1.69)	(0.36,0.73)	

^a^Model 1. Adjusted for sex, age and education (high school or higher vs. less than high school)

^b^Model 2. Further adjusted for smoking (current, former, never), drinking (yes, no), high-fat diet (a little or no, greater than or equal to 25 g/day), vegetables and fruits (a little or no, greater than or equal to 500 g/day), tea (yes, no)

## Discussion

Vitamin D deficiency and insufficiency has become a popular public health problem in the word [26]. Our study was actualized in China, a countryside with mean serum vitamin D levels (28.71 ng/mL). In our cross-sectional study, low serum 25(OH) D3 levels were associated with higher prevalence of dyslipidemia. Compared to the dyslipidemia, participants in the normal serum lipids group had a higher level of serum 25(OH) D3 by 5.55 ng/mL. These discovery is clinically and epidemiologically important. Previous studies had proved that vitamin D deficiency was a risk factor for cardiovascular disease [27], type 2 diabetes [28–30], blood pressure [31] and obesity [32]. The serum lipids status is the major risk factors for dyslipidemias. Our results showed that serum 25(OH) D3 levels had a positive association with HDL-C in the whole crowd and negative correlation with LDL-C was discovered only in normal serum lipid group. These outcome confirmed that serum vitamin D levels correlate with serum lipids in previous studies [33]. These studies suggest that there is a correlation between vitamin D deficiency and the increasing serum levels of TC and LDL-C, as well as the decreasing serum levels of HDL-C [15–20]. However, the conclusions are inconsistent and relationships often disappeared after adjusting for confounding factors [17, 18, 20]. With increasing serum 25(OH)D3 levels, a significant increase was observed in a cross-sectional study [34], but a significant decrease was found in a 13,331 subjects [35]. Furthermore, these study both made a similar adjustments for confounders and have a similar male/female ratio. In our study, no correlation between serum 25(OH) D3 levels and TC were observed and the relationship between LDL-C and serum 25(OH)D3 levels was different in normal serum lipid group and dyslipidemia group. The reason for this is not clear, and further research is needed.

In our study we found that the prevalence of dyslipidemia was different in females and males. The reason why the prevalence of dyslipidemia was different in females and males is unknown. Possibly, the sex differences in life style such as physical activity, smoking, drinking, fat intake and sun exposure act as confounders. In addition, the hormone and the sensitivity of hormone receptor were different in males and females. This may affect the metabolism of lipids. We also found that participants with dyslipidemia were more likely to be higher fat intake. This result is consistent with previous finding that a large amount of fatty acid intake affected the metabolism of blood lipids, causing higher cholesterol, higher triglyceride, higher low density lipoprotein and lower high density lipoprotein [36]. At present, the mechanisms of the correlation between vitamin D and serum lipids are unknown. Vitamin D could affect serum lipids through affecting calcium absorption. Previous study has suggested an increased calcium level may reduce formation and secretion of hepatic TG [37] and intestinal absorption of fatty acid. In addition, calcium could reduce the status of cholesterol through promote the secretion of bile acids [38]. Other studies have provided that vitamin D levels may have an effect on β-cell function and insulin sensitivity which could affect lipid metabolism [39]. The vitamin D levels of personal may be a marker of cardiovascular health [40], but whether vitamin D supplementation can distinct ameliorate cardiovascular status is still unknown. Previous meta-analysis assessed the relationships of vitamin D supplements on serum lipids and demonstrated that vitamin D supplementation could affect LDL-C status, but no significant contact with TC and HDL-C were founded [41]. Results from the research of Alexandra et al. showed that vitamin D supplementation were significantly correlated with serum lipids after adjusting for confounding factors [33]. However, another study showed that the supplementation and treatment with vitamin D were not significantly correlated with plasma adipokine concentrations [21].

The strengths of our study contained the strict sampling design and the rigorous research protocols in demographic data collection and laboratory assays. Few research have study the rural population, which accounts for the majority of the Chinese population. Our results can provide a scientific basis for the prevention of dyslipidemia. Therefore, the samples and results of the study were representative. However, several limitations need to be considered in our study: Firstly, the cross-sectional design assessed a temporal relationship between vitamin D and serum lipids. Secondly, pathophysiological changes of dyslipidemia may affect serum 25(OH) D3 levels or participants with dyslipidemia may modify their life behaviors, containing vitamin D intake. Thirdly, statistical analyses can only adjust for known confounding factors, and can’t adjust for unknown and unmeasured confounding factors. Fourthly, these participants were only come from one province, which may not well represent the Chinese rural population. Fifth, the number of patients are small and the novelty needs to be further improved. Also, dietary information in our study was collected through 24-h recall, these may be inexactitude to assess the eating habits of individuals.

## Conclusions

In summary, low serum 25(OH) D3 levels were correlated with higher prevalence of dyslipidemia in a sample of the rural population in China aged 18–79 years. Serum 25(OH) D3 concentrations were associated with the serum lipids and the associated was different in normal serum lipid group and dyslipidemia group. With the increase of serum 25(OH) D3 levels, the prevalence of dyslipidemia decreased. All of which provide some evidence for vitamin D levels as marker in dyslipidemia prediction. These discovery call for a thorough estimate of the benefits and risks associated with vitamin D levels in the China.

## Abbreviations

25(OH)D3: 25-hydroxyvitamin D3
BMI: Body mass index
CVD.: Cardiovascular disease
HDL-C: HDL cholesterol
LDL-C: LDL cholesterol
TC: Total cholesterol
TG: Triglyceride
